# Molecular characterization by using 12SrRNA and Cytochrome b for identification of species of genus *Ratufa* (Rodentia: Scuiridae) including *Ratufa indica*, endemic species of India

**DOI:** 10.1080/23802359.2019.1667270

**Published:** 2019-10-17

**Authors:** Archana Bahuguna, Ashutosh Singh

**Affiliations:** a Northern Regional Center, Zoological Survey of India, Dehra Dun, Uttarakhand, India;; b Indian Institute of Technology, Mandi, Himachal Pradesh, India

**Keywords:** *Ratufa indica*, *Ratufa bicolour*, *Ratufa macroura*, 12SrRNA, Cytochrome b

## Abstract

Oriental giant squirrels are tree squirrels classified under family Ratufinae. In India, there are three species of genus *Ratufa,* i.e. *Ratufa bicolor*, *Ratufa macroura* and *Ratufa indica*. They are also distributed in South and Southeast Asia. However *Ratufa indica* is endemic to India. The fourth species *Ratufa affinis* is restricted to Maritime Southeast Asia (East Malaysia, Indonesia, Brunei and Thailand) and probably in Singapore. The species is near threatened .The species *R.macroura* is endemic to South Asia. Forests of South and Southeast Asia are hotspots of squirrel diversity but at the same time they are at a high risk of extinction because of high deforestation rate and habitat fragmentation. The present molecular study is the first study of the species of *Ratufa* for their identification. In this study old taxidermy samples were used for amplification of 12SrRNA and Cytochrome b genes. Maximum likelihood and neighbor-joining methods were used to delineate the species by using MEGA 6 and also for molecular analysis for variable, conserved, parsimony and singleton sites. Similarities between species through BLAST indicated 92.21–89.57% between *R.macroura vs. R. bicolour*; 93.22–90% *Ratufa macroura* vs. *Ratufa indica*; 96–92% *Ratufa indica* vs. *Ratufa bicolour*, 93.88% between *Ratufa affinis* vs. *Ratufa indica*, 93.5% *R. affinis* vs. *R. bicolor*, 90.5%. *R. affinis* vs. *R*. *macroura*. *Ratufa bicolor* is noted to be genetically closer to *R. indica* as inferred by using both markers. The BLAST result indicated that the obtained sequences matched 99–100% with their respective species. It was also noted that *R. bicolor* of Jalpaiguri, West Bengal are genetically closer to that of Bhutan. The study also revealed the evolution of *R. indica* and *R. macroura* from a single population.

## Introduction

The oriental giant squirrel (Genus *Ratufa* Gray, 1867) are-cat sized tree squirrels and are classified under Subfamily Ratufinae Moore 1959 (Corbett and Hill 1992; Thorington Jr. and Hoffmann 2005) in Family Sciuridae Gray, 1821. The genus *Ratufa* is represented by four species (Thorington Jr. and Hoffmann 2005) i.e. *R. affinis* (Raffles, 1821), *R. bicolor* (Sparrman, 1778), *R. macroura* (Pennant 1769) and *R. indica* (Erxleben 1777) in the South & Southeast Asia. *R. bicolour* is distributed from East India to Mainland and Maritime Southeast Asian countries (Molur 2008). The species *R. affinis* is restricted only to the Maritime Southeast Asia (East Malaysia, Indonesia, Brunei, and Thailand) and probably present in Singapore, as no recent sighting had been reported from there (Duckworth et al. 2008).The species is near threatened globally (https://www.iucnredlist.org>species). The species *R. macroura* is endemic to South Asia and is distributed in Tamil Nadu and Kerala states of India and various localities in Sri Lanka (Srinivasulu et al. 2004). The last species of the genus, i.e. *R. indica* is endemic to India and is distributed from Central India to South India (Srinivasulu et al. 2004). The genus *Ratufa* is diurnal, arboreal and its natural habitat is tropical montane evergreen forest, dry deciduous and rainforest. These squirrels often make holes in trees and sometimes construct a large globular drey (during breeding season) for shelter in mid-high canopy of the forest (Srinivasulu et al. 2004; animalia.bio > Indian.giant.squirrel). The earliest classification of Sciuridae was based on skeletal and dental morphology (Moore 1959). Based on immunological studies, Hight et al. (1974) showed that *Ratufa* is different from groups such as *Callosciurus, Sundasciurus, Tamiops, Funambulus,* and *Mentes,* which are closely related.

Koprowski and Nandini (2008) reported that the forest of South & Southeast Asia are a hotspot of squirrels diversity but are also at a high risk of extinction because of high deforestation rate and habitat fragmentation. Habitat fragmentation has been considered as the main factor for biodiversity loss (Meffe and Carroll 1997; Battista 2008). In spite of hotspot of squirrel diversity, very few studies have been done on squirrels in the area (Koprowski and Nandini 2008). According to IUCN, the population of species of this genus is continuously diminishing. *R. indica* and *R. bicolor* are listed under Schedule II, *R. macroura* is listed under Schedule I of Indian Wildlife Protection Act, 1972 (Bahuguna and Singh 2013). *R. indica* is also listed in Appendices II of Convention on International Trade in Endangered Species of wild fauna and flora (CITES); as vulnerable (VU) nationally and Data Deficient (DD) globally under Conservation Assessment and Management Plan (CAMP). *Ratufa macroura* is listed as Endangered (EN) under Red Data Book; Endangered (EN) nationally and Data Deficient (DD) globally under CAMP; the species is also listed in Appendices II of CITES. *R. bicolor* is listed in Appendices II of CITES; Vulnerable (VU) nationally and Data Deficient (DD) globally under CAMP.

The genus is facing many anthropogenic threats like agro-industry farming, shifting (Jhum) agriculture practices, human settlement, forest fire & logging (Molur 2008). Apart from these mentioned threats to the genus, hunting (Wang et al. 1989; Evans et al. 2000; Duckworth et al. (1999) and poaching (Srinivas et al. 2008; Bahuguna and Singh 2013; Senthilkumar et al. 2013 and Menon 2003) had also been reported as a reason behind the declining population of the genus. The objective of the study is to do molecular characterization with preliminary data for identification of the species (for wildlife forensic and ecological study).

Mitochondrial genes have been successfully used in determining the interspecies phylogeny in a wide range of taxa (Oshida et al. 2000, 2004; Yu et al. 2004, 2006, 2014, Li et al. 2013, Arbogast 1999) and to evaluate the level of genetic variation within the genus (Hale et al. 2004; Ochoa et al. 2012). Two nuclear genes (c-myc and RAG1) comprising approximately 4500 bp of data (most in exons) are applied for the first time to rodent phylogenetics by Steppan et al. 2004. The study refuted the conventional elevation of the flying squirrels (Pteromyinae) to subfamily status. The study revealed that flying squirrels are derived from one of the tree squirrel lineages. But they used only two gene sequences of *Ratufa* and two species of *Ratufa* i.e *Ratufa bicolor* and one sequence of unknown species of *Ratufa.* However, the present study is the first attempt to construct the phylogenetic relationship of *Ratufa* species (*R. affinis, R. bicolor, R. macroura* and *R. indica*) using the mitochondrial 12 s rRNA and Cytochrome b partial sequences.

## Material and methods

### Sample collection

**Figure 1.**
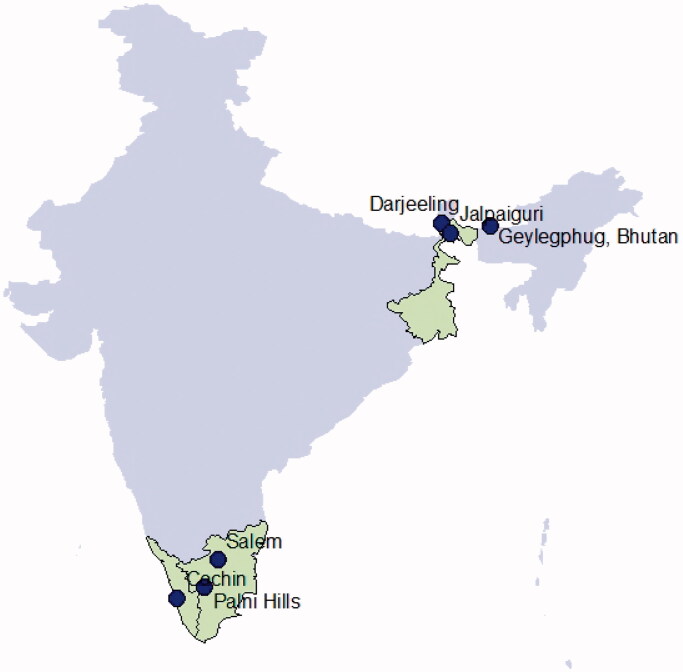
Sample collection sites of species of *Ratufa*.

In this study, taxidermy skin samples (0.5 cm × 0.5 cm) were used from the mammals section of Zoological Survey of India, Kolkata (Table 1, Figure 1). Additionally, 12 s rRNA and Cytochrome b available sequences of species *Ratufa affinis* were obtained from GenBank (Table 1).

**Table 1.** Source of sample collected, sample ID, registration Nos. their locality.

			Accession number	
Species (Common name)	Sample	Locality	12s rRNA	Cytochrome b
*Ratufa macroura* (Grizzled giant squirrel)	*Ratufa macroura*	Salem, Tamil Nadu, India	KP144800*	KP174718*
	*Ratufa macroura*	Palni Hills, Tamil Nadu, India	KP144801*	KP174719*
	*Ratufa macroura*	Salem, Tamil Nadu, India	KP973550*	KP973567*
*Ratufa bicolor* (Black giant squirrel)	*Ratufa bicolor*	Darjeeling, West Bengal, India	KP973557*	KP174716*
	*Ratufa bicolor*	Jalpaiguri, West Bengal, India	KP973558*	KP174717*
	*Ratufa bicolor*	Geylegphug, Bhutan	KP973559*	–
	*Ratufa bicolor*		AY227548	–
*Ratufa indica* (Indian giant squirrel)	*Ratufa indica*	Cochin, Kerala, India	KP973551*	KP973568*
*Ratufa affinis* (Cream coloured giant squirrel)	*Ratufa affinis*	NCBI	AY227547	–
*Rattus norvegicus*#(Brown rat)	*Rattus norvegicus*	NCBI	AY012115	AB033713

*Novel data generated in this study; #Outgroup (Oshida et al. 1996).

### DNA isolation, PCR amplification, and sequencing

The skin samples thus collected were cleaned with MilliQ water and hydrated before digestion by incubating the dried skin sample for 24 h in 1 ml TE solution (Tris 10 mM and EDTA 1 mM, pH 7.6) (Barros and Morgante 2007). After 24 h of hydration, the DNA was isolated from skin sample using HiPur ATM Forensic Sample Genomic DNA Purification Kit (HIMEDIA).12s rRNA sequences were amplified using a set of primer pair, L1091 and H1478, and a primer set of L14841 and H15149 was used to amplify Cytochrome b gene (Kocher et al. 1989). The PCR reaction was performed in Q-cycler, Quanta Biotech (Surrey, UK), in a total volume of 25 µl of reaction mixture (10 mM PCR buffer-with MgCl_2_, 2.5 ml; 10 mM dNTP’s,2.5 ml; 5 pmol primer, 0.45 ml each; 15 ng of DNA template;1.5 U Taq enzyme).

Polymerase chain reaction consisted of initial denaturation of 94 °C for four minutes and each cycle of denaturation for 1 min at 94 °C, hybridization for 1 min at 55 °C (50 °C for Cytochrome b) and extension for 1 min at 72 °C followed by final elongation for 10 min at 72 °C. The cycle was repeated for 35 times. The PCR products were sequenced using ABI's AmpliTaq FS dye terminator cycle sequencing chemistry on an automated ABI 3100 Genetic Analyser. All experiments were performed in a PCR workstation (Bangalore GeNeiTM). Negative controls were used in all DNA extraction and PCR amplification to control for potential contamination. 12SrRNA gene and Cytochrome b gene sequences thus generated are submitted to NCBI after conducting sequence alignment by Bioedit and by checking their similarity with species of genus *Ratufa*.

## Data analysis

Sequences were visualized and edited using Chromas 1.6 (Technelysium Pty Ltd., South Brisbane, Australia). To crosscheck, quarry sequences were compared using GenBank BLAST (http://www.ncbi.nlm.nih.gov/BLAST). Percent similarities between species through BLAST indicated 92.21–89.57% between *R. macroura vs. R. bicolour*; 93.22–90% *Ratufa macroura* vs. *Ratufa indica*; 96–92% *Ratufa indica* vs. *Ratufa bicolour*, 93.88% between *Ratufa affinis* vs. *Ratufa indica*, 93.5% *R. affinis* vs. *R. bicolor*, 90.5%. *R.affinis* vs. *R*. *macroura*. *Ratufa bicolor* is noted to be genetically closer to *R. indica* as inferred by using both markers. CLUSTAL W was used to compare DNA sequences and for alignment of genetic data by using BioEdit v 7.0.9.0 software (Hall 1990) with outgroup *Rattus norvegicus* (for 12SrRNA: AY012115 and for Cytochrome b: AB033713). All sequences were proofread and analyzed by using MEGA6.0 (Tamura et al. 2011) and were aligned by using Clustal W (Thompson et al. 1994). MEGA 6.0 was used for finding the conserved, variable, parsimony informative and singleton sites as well as for phylogeny construction. Two methods were used for phylogeny construction: (i) maximum likelihood and (ii) neighbor-joining with Kimura 2 Parameter (Pevsner 2009). All trees were subjected to bootstrap analysis with 1000 replicates to get bootstrap value support.

## Results

12Sr RNA gene was amplified from extracted DNA of *Ratufa* species i.e. *R. affinis, R. bicolor, R. macroura* and *R. indica*. After sequencing, the unambiguous lengths of 12S rRNA (ca. 385 bp) were obtained and the same was tried for Cytochrome b. The BLAST result indicated that the obtained sequences matched 99–100% with their respective species. In 12srRNA with 385 bp, nine haplotypes, 289 conserved sites, 95 variable sites, 40 parsimony informative sites and 55 singleton sites were obtained.

### Species specific sites in 12S rRNA gene

In relation to the mitochondrial gene of *Rattus norvegicus* out of 385 bp of 12SrRNA gene, there were 320,309, 308 and 309 conserved sites, 75, 73, 65, and 65 variable sites and 7, 2, 0 and 0 parsimony informative sites were observed in *Ratufa macroura, Ratufa bicolor, Ratufa indica* and *Ratufa affinis* respectively. 68 singleton, 70 singleton sites were observed in *Ratufa macroura, Ratufa bicolor* respectively. The nucleotide composition of the entire sequence region of 12S rRNA gene (385 bp) is A 34.5%, T 23.8%, C 23.8%, and G 18.00%.the estimated Transition/Tranversion bias is 2.78.

### Species specific sites in Cytochrome b gene

Genetic analysis was done by MEGA 6 with seven sequences, ca. 359 bp with *Rattus norvegicus* as outgroup inferred 77 variable/polymorphic (segregating) sites and seven haplotypes (including *Rattus norvegicus*). In relation to the mitochondrial gene Cytochrome b of ca 359 bp of outgroup *Rattus norvegicus* as determined by MEGA 6, there were 301, 300 and 296 conserved sites; 58, 57 and 63 variable sites and 0,4 and 0 parsimony informative sites in *Ratufa bicolor, Ratufa macroura* and *Ratufa indica*, respectively. These sites can be used to differentiate the species of *Ratufa*. There were 58, 53 and 0 singleton in *Ratufa bicolor, Ratufa macroura* and *Ratufa indica*, respectively. Seven haplotypes were present. The nucleotide compositions of the entire sequenced region of Cytb (bp) are: 26.6% T, 29.8% C, 28.2% A and 15.5% G and the estimated Transition/Tranversion bias is 1.64.

## Discussion

### Molecular characterization and phylogeny of species of *ratufa*

**Figure 2.**
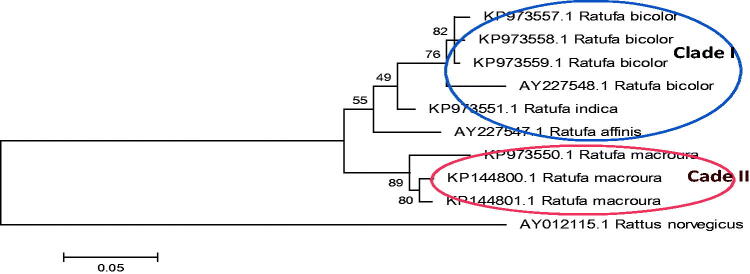
Phylogenetic analysis using 10 gene sequences of 12SrRNA, with maximum likelihood algorithm. The tree depicts that *Ratufa indica* is forming a sister clade with *Ratufa bicolor*. There are two major clades in tree.

**Figure 3.**
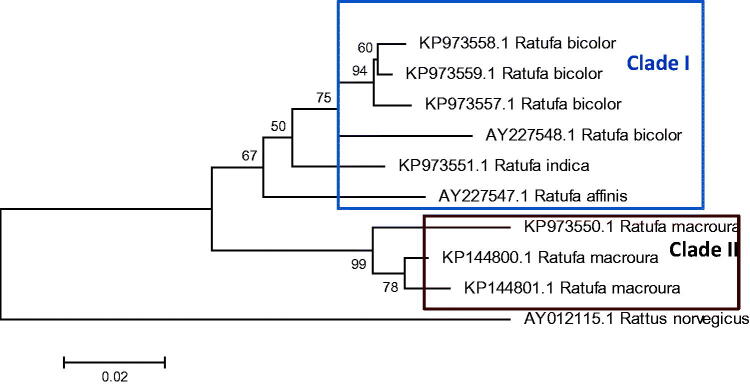
Phylogenetic analysis using 10 gene sequences of 12SrRNA, with neighbour joining algorithm. The tree depicts that *Ratufa indica* is forming a sister clade with *Ratufa bicolour* with two major clades in tree.

**Figure 4.**
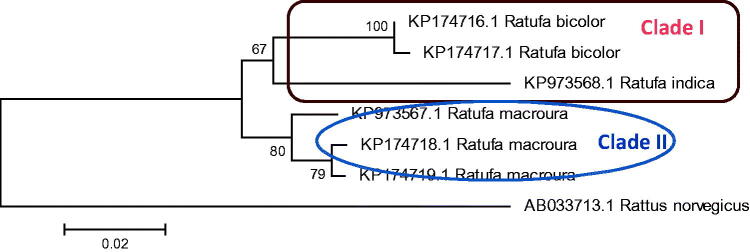
Phylogenetic analysis using 6 gene sequences of Cytochrome b gene, with neighbour joining algorithm. The tree depicts that *Ratufa indica* is forming a sister clade with *Ratufa bicolour* with two major clades in tree.

**Figure 5.**
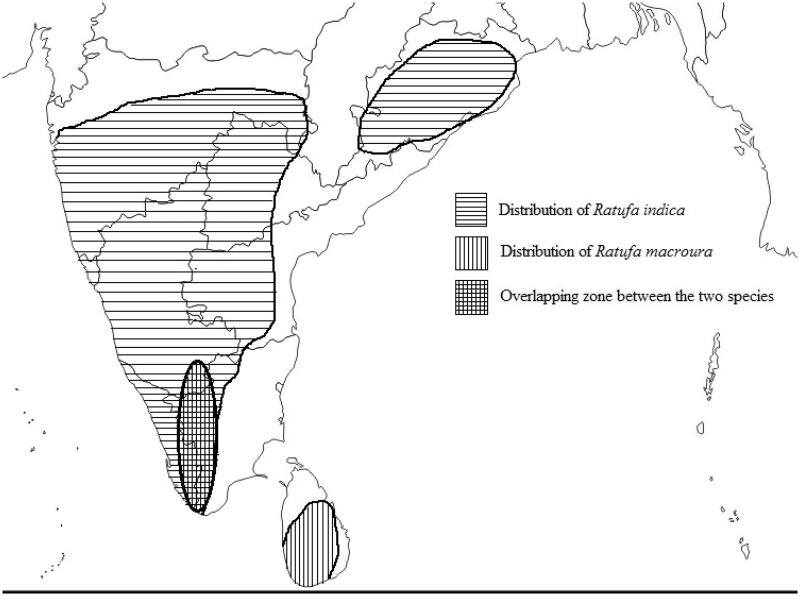
Distribution of species of *Ratufa indica*, *Ratufa macroura* and overlapping zone between two species based on literature (source: prepared by Ashutosh Singh).

The *Ratufa* species are distributed in different zoogeographic sub-regions of Southeast Asia (Moore 1960; Molur et al. 2005; Thorington Jr. and Hoffmann 2005). The genus *Ratufa bicolor* is widespread in South Asia, Southeast Asia, and southern China. In South Asia, the species has been reported from Bangladesh, Bhutan, eastern India and Nepal (Molur et al. 2005). In Southeast Asia, the species has been widely distributed in Myanmar, Thailand, Cambodia, Vietnam, Malaysia, and Indonesia. In China, the species has been reported from southern Yunan, southern Guanxi, eastern Xizang and Hainan Island (Smith and Xie 2008). In the present study, phylogeny obtained from 12 S rRNA sequence (Figures 2 and 3) shows *R. bicolor* as a sister group to all other species of the genus except *Ratufa macroura*. In Cytochrome b gene phylogeny (Figure 4) *R bicolor* shows close relationship with *R. indica*. The hybridization between the two, *Ratufa indica* and *Ratufa macroura* had been reported previously by some researchers (Corbett and Hill 1992; Joshua 1996). Interestingly, the overlapping distribution was also reported in the species and thus must be having overlapping habitat (Figure 5) and as a result interbreeding between the two species has been reported by Joshua 1996, but more sampling is required to prove hybridization.

**Figure 6.**
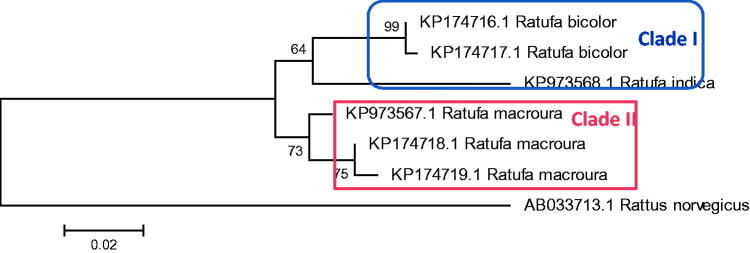
Phylogenetic analysis using 6 gene sequences of 12SrRNA, with maximum likelihood algorithm. The tree depicts that *Ratufa indica* is forming a sister clade with *Ratufa bicolour* with two major clades in tree.

**Figure 7.**
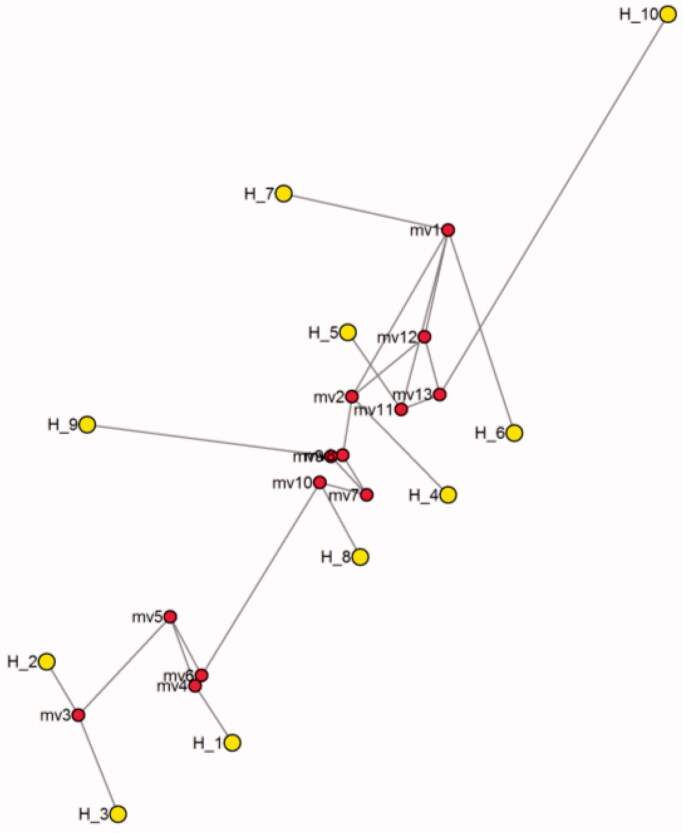
Median joining network by using 12SrRNA gene of species of genus *Ratufa*, haplotype 1, 2, 3 belongs to *Ratufa macroura,* haplotypes 4, 5, 6, 7 *Ratufa bicolour,* haplotypes 8 to *Ratufa indica*, 9 to *Ratufa affinis* and haplotype 10 to outgroup *Rattus norvegicus*.

**Figure 8.**
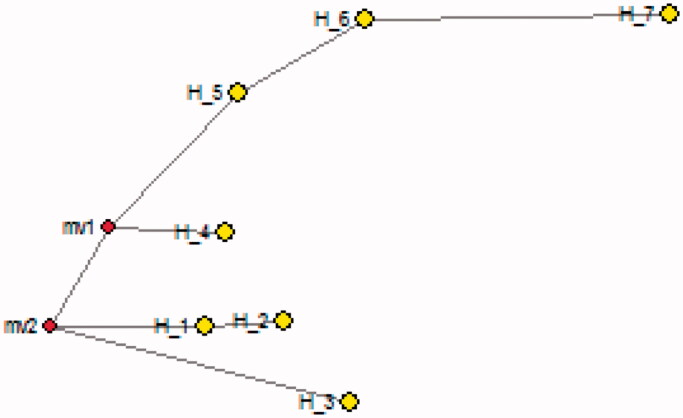
Median joining network by using Cytochrome b gene of species of genus *Ratufa*, Haplotype 1, 2 belongs to *Ratufa bicolour*, haplotypes 3 *Ratufa indica* and haplotype 7 to outgroup *Rattus norvegicus*.

However the various sites as reported with reference to *Rattus norvegicus* i.e. out of 385 bp of 12SrRNA gene, there were 320,309, 308 and 309 conserved sites, 75, 73, 65, and 65 variable sites and 7, 2, 0 and 0 parsimony informative sites and 68, 70,0 and 0 singleton sites were observed in *Ratufa macroura, Ratufa bicolor, Ratufa indica* and *Ratufa affinis* respectively are species specific and are useful in species identification for wildlife forensic and prey predator relationship (Table 2). Similarly in case of Cytochrome b with 359 bp with *Rattus norvegicus* as outgroup inferred 77 variable/polymorphic (segregating) sites and six haplotypes. In relation to the mitochondrial gene Cytochrome b of ca 359 bp of outgroup *Rattus norvegicus* as determined by MEGA 6, there were 301, 300 and 296 conserved sites; 58, 57 and 63 variable sites and 0, 4 and 0 parsimony informative sites and 58, 53 and 0 singleton sites in *Ratufa bicolor, Ratufa macroura* and *Ratufa indica*, respectively can be used to differentiate the species of *Ratufa* (Table 2).

**Table 2.** Species specific sites as inferred by two markers: 12srRNA and Cytochrome b gene analysis (using MEGA 6).

Species	Conserved sites	Variable sites	Parsimony informative sites	Singlton
Species specific sites as inferred by 12SrRNA gene analysis (using MEGA 6)				
* Ratufa macroura*	320	75	7	68
* Ratufa bicolor*	309	73	2	70
* Ratufa indica*	308	65	0	0
* Ratufa affinis*	309	65	0	0
Species specific sites as inferred by Cytochrome b gene analysis (using MEGA 6)				
* Ratufa macroura*	301	58	0	58
* Ratufa bicolor*	300	57	4	53
* Ratufa indica*	296	63	0	0
* Ratufa affinis*	Gene sequence not available			

In the present study, samples of *Ratufa macroura* are clustered in a single clade. These samples were collected from Tamil Nadu state of southern India. *Ratufa indica* forms a sister clade with *Ratufa bicolor*. The two species *macroura* and *indica* are restricted to India and Sri Lanka, whereas *R. indica* is endemic to India. It was also noted that the two species shared certain characters like 1) the crown and nape are separated by a contrasting colour mark crosses the top of head between the ears, 2) a dark, narrow stripe extending from the ear down to the side of the head. The distribution of *Ratufa* is also known as a zoogeographic puzzle since the species are found on both sides of the Garo-Rajmahal gap. The gap has proved to be a barrier for other squirrels, such as *Funambulus*, which do not occur east of the gap, and *Callosciurus*, which do not occur west of the gap (Thorington and Cifelli 1990). The distribution and origins of *Ratufa* may be more ancient than that of the other two genera. According to Thorington and Cifelli 1990, certain peculiar anatomical and morphological characters make arboreal gigantism possible in these squirrels. The presence or absence of giant squirrels could be useful in assessing habitat quality (as indicator species), because of their ecological requirements, dependence on canopy continuity, and on fruits and seeds for food.

According to Hight et al. 1974, tree squirrels have evolved from the genus *Protosciurus* which existed during the Oligocene epoch in North America and migrated into Europe through Asia. Although there is fossil evidence of *Ratufa* from Central Europe, its present range is restricted to the Oriental zoogeographical region (Hight et al. 1974). According to Emry and Thorington Jr. 1982, the divergence of tree squirrels might have been possible from the *Sciurus* squirrels of North America during the mid-miocene period, i.e. before the establishment of the land connection between North and South America. Thus the distribution of *Ratufa macroura* might have occurred in late Miocene from India to Sri Lanka during which the two landmasses reconnected and severed many times due to the rising sea levels. But *Ratufa indica* was not able to cross the major landmass of India and remain restricted in distribution to India.

However, based on pelage colour, Abdulali and Daniel (1952) reported eight races of *Ratufa indica*. Agarwal and Chakraborty (1979) described seven subspecies of *Ratufa* based on the collection of Zoological Survey of India. An endemic race of the Indian giant squirrel, *Ratufa indica dealbata*, originally restricted to the Surat Dangs, is reported to be extinct as a result of the depletion of their natural habitats.

Peninsular India and Sri Lanka, which were land fragments that separated from Madagascar about 90 million years ago and merged with Asia, have shared a large part of their geological history. The first separation of India and Sri Lanka happened in the early Miocene era, roughly 15 million years ago. Since then, the two landmasses have reconnected and severed many times, most recently about 10,000 years ago, due to the rising sea levels. This separation has allowed animals in Sri Lanka to become unique and evolve independently, in a process called endemism. Various study suggests that the evolution of these species in Sri Lanka is a result of their separation from their Indian counterparts when the sea levels rose in the Miocene era like in case of some gecko species like *H. scabriceps* (scaly gecko) and *H. lankae* (Sri Lankan leaf-toed gecko) that inhabit the semiarid open habitats survived in both India and Sri Lanka. The Indian subcontinent underwent a drastic change in vegetation during the late Miocene era where some forests gave way to open grassland habitats. Although these changes caused the extinction of wetland adapted animals like caecilians and shield-tail snakes, those that were adapted to open habitats, like the *Sitana* lizards, survived and formed new species. The land bridge between India and Sri Lanka was re-established around this period, allowing for a faunal exchange (Thorington and Cifelli 1990).

## Conclusion

The genus *Ratufa* occurs in Indo-Malayan regions and these giant squirrels are found in diverse habitats ranging from deciduous to evergreen forests. All the four species of giant squirrels of the Oriental region belong to the genus *Ratufa*. They are facing a threat to survival because of habitat fragmentation and poaching. Thus the present molecular study based on two markers 12SrRNA and Cytochrome b is useful in providing species specific molecular sites for identification of the species. The phylogenetic study also provides delineation of species of the genus and also indicates that *R. bicolor* is genetically closer to *R. indica* as they are present in the same clade and *R. macroura* formed the separate clade. The present study also revealed that *Ratufa bicolour* from Jalpaiguri, West Bengal, is genetically closer to that of from Bhutan. The geographical isolation among the population of *Ratufa* during miocene might have led to the evolution of species *Ratufa macroura* (species endemic to India and Sri Lanka) and *Ratufa indica* (species endemic to India).
